# Effects of Cognitive Exercise Therapy on Upper Extremity Sensorimotor Function and Activities of Daily Living in Patients with Chronic Stroke: A Randomized Controlled Trial

**DOI:** 10.3390/healthcare10030429

**Published:** 2022-02-24

**Authors:** Wonho Choi

**Affiliations:** Department of Physical Therapy, Gachon University, Incheon 21936, Korea; whchoi@gachon.ac.kr; Tel.: +82-32-820-4423; Fax: +82-32-820-4420

**Keywords:** cognitive exercise therapy, stroke, sensorimotor, activity of daily living

## Abstract

This study investigated the effects of cognitive exercise therapy on upper extremity sensorimotor function and daily activity in patients with chronic stroke and compared these effects to those of conventional occupational therapy. The 30 patients with chronic stroke (mean age: 63.6 ± 12.7 years; height: 162.8 ± 8.1 cm; weight: 60.6 ± 7.6 kg; body mass index: 22.8 ± 1.9 kg/m^2^) were divided into two treatment groups with 15 patients in each. The respective interventions were provided for 30 min per day, five times weekly for 4 weeks. Manual and sensory function tests were conducted to evaluate the sensorimotor function, while the Korean-Modified Barthel Index was used to assess daily activities. All outcome variables were assessed before and after the interventions. A significant interaction was observed in sensory function (*p* = 0.001) but not motor function or daily activities (*p* > 0.05). No significant main group effects were found for any outcome variables (*p* > 0.05). The experimental group showed significant improvements in motor function (*p* < 0.001), sensory function (*p* < 0.001), and daily life activities (*p* = 0.001) after cognitive exercise therapy, whereas the control group showed significant improvement only in daily life activities post-intervention (*p* = 0.012). These results demonstrated the positive effects of cognitive exercise therapy on upper extremity sensorimotor function and daily life activities and the lack of improvement in motor and sensory function following conventional occupational therapy in patients with chronic stroke. Thus, the combination of cognitive exercise and conventional occupational therapies may be an effective way to improve sensory function and upper extremity motor function in patients with chronic stroke.

## 1. Introduction

A stroke, a sudden impairment of body function caused by a blockage of blood flow to the brain, has the third-highest mortality rate after cancer and heart disease [1,2]. More than 85% of patients with stroke experience hemiplegia, and 55–75% of these patients have upper extremity impairment [3]. Impaired upper extremity function may result in decreased mobility of the shoulder joint, muscle weakness, sensory impairment, spasticity, and lack of coordination [4]. Subsequently, these lead to sequelae such as limited joint movement and limited upper extremity function [5]. Sensory impairment occurs in at least 50% of stroke patients and is expected to be higher with accurate examinations [6]. Sensory impairment interferes with correct movement and sharply reduces movement based on sensory input or feedback [7]. Patients with sensory impairment avoid movement, and their movements are dull and uncoordinated [7,8]. Thus, for patients with sensory impairment, proprioception, tactile sensation, pressure sensation, and stereoscopic sensation are essential for smooth movement by activating natural movements [9]. As these sensations are closely related to functional recovery after stroke, the impaired sensory function is one of the most significant factors hindering rehabilitation in patients with stroke. Additionally, impairment of upper extremity and sensory functions reduce the performance of basic daily activities. Therefore, patients cannot perform these activities independently and rely on the help of their caregivers [10].

Many interventions have been applied to address upper extremity function in patients with stroke. Among these, task-oriented upper extremity training improved the function of affected upper extremities [11,12,13]. The complex interaction between the left and right brains during bilateral tasks of the upper extremities enhanced the function of the affected upper extremity [11,12]. Moreover, constraint-induced movement therapy in the affected upper extremity of patients with stroke improved upper extremity function [13]. However, these interventions are therapeutically accessible only to patients with at least minimal voluntary motor skills. Another intervention is mirror therapy, which reorganizes brain areas along with physical rehabilitation. Based on the theoretical mirror neuron system, this intervention method promotes functional recovery of the upper extremities by inducing the recovery of motor function and movement on the affected side [14]. Another invention is mental practice, which allows patients to acquire and improve motor skills through thoughts of certain movements rather than actual body movements [15]. When a certain level of sensory recovery can be expected, repeated stimulation may provide necessary sensory feedback; moreover, the senses may be improved to a conscious level by focusing on a given sense [16].

Cognitive exercise therapy is a therapeutic intervention that aims to reorganize the central nervous system through learning motor function recovery [17,18]. This therapy emphasizes the close relationship of motor function to the activation of cognitive processes in the brain, such as perception, attention, memory, judgment, and language. The quality of recovery depends on the correct identification of the cognitive factors [19]. Cognitive exercise therapy allows various movements or actions to be performed through cognitive training processes, rather than movement training through interaction between the body and environment, to build a brain schema with four principles. The first principle is ‘attention’, which focuses on enhancing the effectiveness of treatment and reorganizing exercises in the treatment process. Second, patient treatment requires attention to somatosensory information with the eyes closed. Third, specific treatment instruments or tools are used to treat cognitive problems through interactions between the body and the environment. Finally, cognitive exercise does not force patients to conduct muscle contractions to accurately mobilize motor units. As such, the purpose of cognitive exercise therapy is not to teach patient-specific body postures but rather to develop and maximize the ability to organize the spatial, temporal, and intensity factors of the exercise sequence in the interaction between the body and environment [20].

Studies on cognitive exercise therapy in patients with stroke have shown improved upper extremity functions such as motor function, manual skills, and strength through image training of cognitive exercise therapy [21]. A comparison of cognitive exercise profiles showed improvements in sensory recognition and movement in the paralyzed arm [22]. In another study, activities of daily living (ADL) and motor function of the upper extremity improved after cognitive exercise therapy through contact tasks using a sponge on the paralyzed arm and spatial tasks using a graphic panel in patients with acute stroke. In addition, cognitive exercise profiling improved joint angle, spatiality, and shape recognition in recognition patterns [23].

However, most studies on cognitive exercise therapy were single-case or single-group studies with non-randomized groups and without a control group. Thus, these effects have not been directly compared. In addition, while various assessments have been conducted on upper extremity functions and daily activities, changes in cognitive patterns and pathological characteristics were described rather than used to directly assess sensory function recovery, which is a key factor in cognitive exercise therapy. While this approach is suitable for a single case study or qualitative research with a small number of subjects, it is difficult to quantify the objective results. Therefore, this study evaluated the effects of cognitive exercise therapy and compared them to conventional occupational therapy on upper extremity motor function, sensory function, and daily activities in patients with chronic stroke. We hypothesized that cognitive exercise therapy would improve upper extremity motor and sensory function and daily activities compared to conventional occupational therapy.

## 2. Materials and Methods

### 2.1. Ethical Approval

The study was conducted according to the guidelines of the Declaration of Helsinki and was approved by the Gachon University Institutional Review Board (IRB, 1044396-201804-HR-097-01). All participants signed a statement providing their informed consent before beginning the study. This trial was not registered. All procedures were conducted as approved by the IRB, and there was no selective outcome reporting.

### 2.2. Participants

A total of 37 patients with chronic stroke voluntarily participated in the study; among these, seven patients who did not meet the inclusion criteria were excluded. Thus, this study included 30 patients.

The inclusion criteria of the study participants were: patients diagnosed with hemiplegia due to stroke at 6 months after onset. The following types of patients were excluded: patients with cognitive impairment or dementia with a Korean version of mini-mental state examination (K-MMSE) score of 19 or higher who could understand and follow the instructions of the therapist; patients with Brunnstrom upper limb recovery stage 2 or higher; patients with a modified Ashworth scale stiffness level of 1+ or lower; patients with auditory and visual impairment or visual field defects; and patients with severe contracture due to orthopedic disease of the shoulder, elbow, and wrist joints.

### 2.3. Procedure

The 30 patients were randomly assigned to the control, and experimental groups administered conventional occupational and cognitive exercise therapies, respectively, with 15 patients assigned to each group using simple randomization methods that were independently conducted. Concealed allocation was performed using a computer-generated randomized table of numbers before data collection.

**Figure 1:**
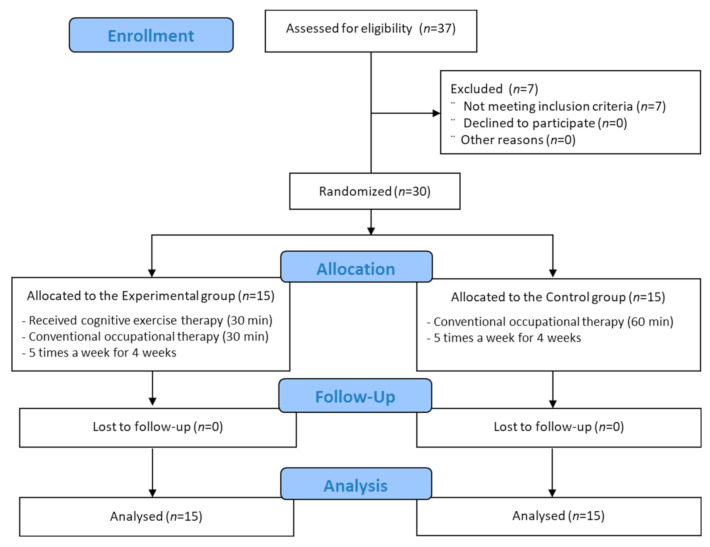
Flow diagram.

All participants in both groups underwent a manual function test (MFT), sensory function test (SFT), and Korean version of the modified Bathel index (K-MBI) evaluation for baseline assessment. The control group underwent conventional occupational therapy for 30 min twice daily, five times weekly, for 4 weeks (a total of 20 times) with the help of an experienced occupational therapist. The experimental group received conventional occupational therapy for 30 min per session, five times weekly, as well as cognitive exercise therapy for 30 min per session, five times a week, for 4 weeks (a total of 20 sessions). Both groups underwent their respective interventions for 30 min each in the morning and 30 min in the afternoon to minimize the physical fatigue (Figure 1).

### 2.4. Measurements

#### 2.4.1. Manual Function Test

The MFT was developed to measure the motor function of the affected upper limb in patients with a stroke [24]. The MFT comprises 32 test items that reflect the recovery of upper limb motor function and functional level of daily life activities. The reliability of the MFT was consistently above 0.95, and the validity of the MFT was good for both the Brunnstrom stage and the Stroke Impairment Assessment Set [25].

#### 2.4.2. Sensory Function Test

The SFT examined seven sensations (pain, tactile, pressure, temperature, kinesthetic, stereognostic, and discriminative senses) in the hemiplegic upper limbs. The total score is 14 points for a total of seven sensations, with participants receiving 0 points they did not recognize both the sensory type and location of stimulation, 1 point for only one of the two, and 2 points for both sensation type and location. A preliminary test was performed three times on the unaffected upper limb before conducting the test for patients to understand the test. Ten tests were conducted on each item, and if more than seven of them were successful, the sensation was considered intact.

#### 2.4.3. Korean-Modified Barthel Index

The K-MBI, which consists of 10 items describing activities of daily living (ADL) and mobility, was scored to measure the degree of assistance required by an individual and was used to assess ADL in patients with stroke [26]. Each item is rated 5-Likert scale, with weights added according to the item. The higher the total score, the more independent on performing ADLs [26]. The reported reliability of the K-MBI was 0.994, with a range of discriminative index of 0.783–0.909 [27].

### 2.5. Intervention

#### 2.5.1. Cognitive Exercise Therapy

Cognitive exercise therapy involves two types of tasks: spatial and tactile. Spatial cognitive tasks involve perceiving elements as direction and distance, while tactile cognitive tasks involve perceiving properties from contact with an object, such as the surface, pressure, friction, and weight. Each participant in this study performed five spatial and tactile tasks. Two or three tasks were selected based on the participants’ recovery levels.

**Figure 2:**
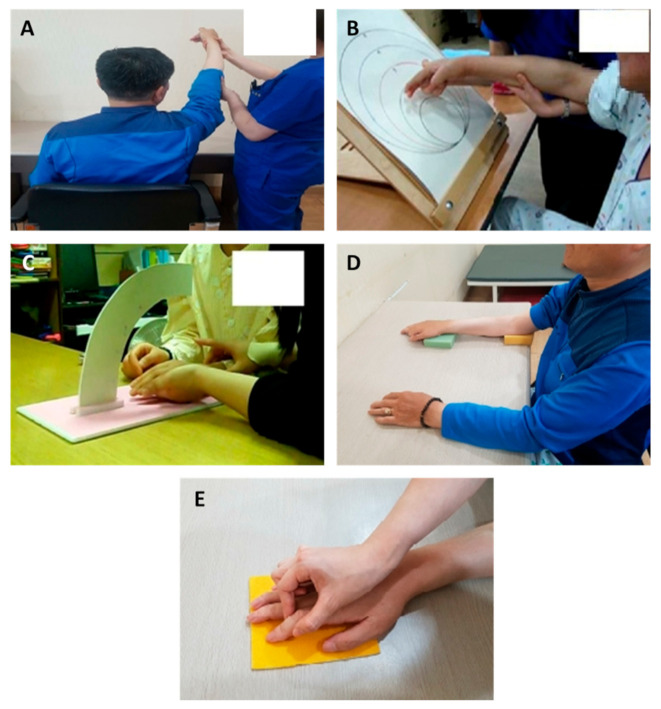
(**A**) Shoulder joint recognition training by motor imagery. (**B**) Shoulder and elbow joint recognition training using a circular track plate. (**C**) Training on awareness of elbow and wrist joint angles using a Bogen. (**D**) Training on pressure awareness of the elbow and wrist using a sponge. (**E**) Finger tactile recognition training using a tactile plate.

Shoulder joint recognition training by motor imagery [28]: the spatial task in shoulder joint recognition training is to control the shoulder joint through sensory images of the joint during spatial tasks. The images help improve recognition movements during bending and opening the shoulder joint. The participants were asked to sit upright on a chair without touching the backrest. After bending and opening the shoulder joint on the unaffected side, the participants were asked to describe the sensation. The therapist passively moved the participant’s shoulder joint on the affected side and asked the participants to describe the sensation again. Then, motor imagery of the unaffected side was used to induce movement of the unaffected side. The participants were asked to describe the sensation of the shoulder joint on the affected side. The feeling of bending and opening of the shoulder joint on the affected side was expressed with active assistance from the therapist. The feelings of the shoulder joint’s movement on the affected and unaffected sides were compared. The participants were then asked to express their sensory images during the movement of the shoulder joint on the unaffected and affected sides. The images were compared, and the differences were expressed in words. This training increased participant awareness of the shoulder joint using the movement image of the shoulder joint (Figure 2A).

Shoulder and elbow joint recognition training using a circular track plate [19]: shoulder and elbow joint recognition training, a cognitive task that uses special senses, was conducted using a circular track plate to distinguish the movement angle of the shoulder joint. In this task, the participants distinguished the distance of each circle of the track place to improve recognition of shoulder joint movement. The participants were asked to sit on a chair without touching the backrest in front of the track plate on a table. The size of each track plate was analyzed visually. After blinding the participant, the therapist supported the participant’s arm to provide passive movement, which provided information on the circle distances and locations. Each participant was given a cognitive task to identify the distance and describe the response in words. After unblinding, the participants’ descriptions were compared to the results of the visual analysis. The participants perceived cognitive tasks to identify distance through movements of the shoulder joint in space (Figure 2B).

Elbow and wrist joint angle awareness training using a Bogen [19]: among spatial tasks, this training on distance identified the movement angle of the elbow joint using a Bogen. This task was conducted to distinguish the distance to each grid line of Bogen and increase awareness of the elbow joint or wrist movement. The Bogen was placed on a table, and the participant was asked to sit in front of the table on a chair without touching the backrest. The participants visually observed the differences in grid lines and were blinded afterward. The elbow joint was then placed on the table, and the therapist supported the patient’s forearm and fingers to help the participant passively identify differences in distance. Afterward, each participant was given a cognitive task to identify distance and was asked to describe their response in words. After unblinding, the participants’ descriptions were compared with the visual analysis results. The task was gradually subdivided using a Bogen with finer grids to adjust the difficulty (Figure 2C).

Elbow and wrist pressure awareness training using a sponge [22]: this tactile task involved the use of the shoulder, elbow, and wrist joints to perceive the pressure differences. Sponges were used to improve the ability to perceive the gradient pressure differences. The participants were asked to sit on a chair without touching the backrest, and three types of sponges of different thicknesses were used. After blinding the participants, the therapist placed a sponge on one area of the body and gently pressed the sponge. The participants were asked to pay attention to the area in which the sponge was placed and to distinguish differences in the hardness of the sponge. During this process, the participant was asked to pay attention to the body part (elbow, shoulder, or wrist) rather than the sponge. The therapist asked for differences in the position and hardness of the sponges. If the participant was able to distinguish the differences in the hardness of the sponge in one area on the unaffected side, the pressure differences between the affected and unaffected sides were assessed. The task was conducted with the participant in a relaxed state, and the task allowed the participant to increase recognition of the body by distinguishing differences in sponge pressure (Figure 2D).

Finger tactile recognition training using a tactile plate [19]: this tactile task involved distinguishing between materials using the fingers and a tactile plate for the recognition of surface materials and perception of finger movements. Each participant was asked to sit on a chair without touching the backrest and to place both arms on a table. Five tactile plates of different soft or rough materials were prepared. Visual and tactile information for both the affected and unaffected sides was provided to the participant. The therapist passively moved the participant’s finger to provide tactile sensations to the plates, and the differences in the surface materials and finger movements were evaluated. The tactile sensation was further tested using plates made of materials with minor differences (Figure 2E).

#### 2.5.2. Conventional Occupational Therapy

The conventional occupational therapy provided in this study was based on the occupational therapy practice framework (OTPF3) [29]. The therapy included passive joint exercises to reduce the spasticity of the upper extremity muscles and joints affected by stroke, as well as active joint exercises such as small muscle activity using different tools, micro-movement training, and two-handed exercises. Neurodevelopmental treatment and pain management were provided according to the patient’s level of recovery. ADL, including wheelchair operation, climbing up and down stairs, functional movement, eating, dressing, and personal hygiene, were assessed. Depending on the level of recovery of the participant, 2–3 tasks were selected and performed.

### 2.6. Sample Size Estimation

The sample size was estimated using G*power 3.1.9.4 (Heinrich Heine University, Dusseldorf, Dusseldorf, Germany). An estimated sample size of 26 participants was obtained using an effect size of 1.03, the alpha error probability of 0.05, and power of 0.80 for the effect of cognitive exercise therapy on upper limb function when a clinically significant difference was observed between two independent means with a one-tailed test. The effect size (Cohen’s d) of 1.03 was calculated from the results of a previous study [20]. An additional 10% of participants were recruited to account for unanticipated attrition.

### 2.7. Statistical Analysis

Statistical analysis was performed using IBM SPSS Statistics for Windows, version 26.0 (IBM Corp., Armonk, NY, USA), and the measured values of all items were calculated as mean and standard deviation (SD). Shapiro–Wilk tests were used to test the normal distribution of the data. Chi-squared and independent t-tests were performed to compare the general participant characteristics using the homogeneity test between groups. Repeated-measures analysis of variance (RM ANOVA) was conducted to assess the group-by-time interaction effect of interest. When a significant interaction was found, a paired t-test was conducted to compare the outcome variables before and after the intervention in each group. The level of statistical significance was set at α = 0.05.

## 3. Results

The general participant characteristics are shown in Table 1. These characteristics did not differ significantly between the groups (*p* > 0.05).

Comparisons of homogeneity between the control and experimental groups before their respective interventions showed no significant differences in MFT, SFT, and K-MBI at baseline (*p* > 0.05).

Table 2 shows the outcome variables before and after the interventions in the cognitive exercise therapy and conventional occupational therapy groups. There was no significant interaction in the mean MFT score between groups and time (*p* = 0.626). The main effect of the group was not significant (*p* = 0.961). The baseline MFT score, 8.40 ± 9.34 points, significantly improved to 9.93 ± 9.53 points post-intervention in the experimental group (*p* < 0.001), while the control group showed no significant improvement (8.80 ± 8.93 to 9.87 ± 9.61, *p* > 0.05).

While a significant group-by-time interaction effect was observed for the mean SFT score (*p* = 0.001), no significant main effect of the group was observed (*p* = 0.738). The SFT improved significantly from 7.07 ± 2.91 points at baseline to 8.87 ± 2.97 points post-intervention in the experimental group (*p* < 0.001). Conventional occupational therapy also showed a significant improvement in SFT (8.20 ± 3.32 8.47 ± 2.88, *p* = 0.104). Subdivision of the SFT score showed no significant interactions for all sensations (*p* > 0.05), except for tactile and kinesthetic senses (*p* < 0.001 and 0.001, respectively).

No significant interaction was observed in the K-MBI score between group and time (*p* = 0.401), and no statistically significant difference was found (*p* > 0.05). Both groups showed significant improvements in daily activity before and after the intervention (57.20 ± 19.27 to 61.47 ± 20.64, *p* = 0.001 and 46.73 ± 22.59 to 53.07 ± 19.42, *p* = 0.012, respectively).

## 4. Discussion

A stroke interrupts the blood supply to the brain and causes bleeding in the brain tissue, leading to various types of neurological damage [30]. Motor weakness of the upper extremities is commonly observed after the stroke, which leads to independence in activities of daily living [31]. This damage affects various sensory elements that detect changes in movement or distinguish the direction of movement of the extremities. Therefore, functional exercises must be developed to increase the use of the upper extremities [20]. Cognitive exercise therapy controls the recovery of motor function in the somatosensory system by reproducing the interaction between the body and the environment in the cranial nerves and organizing activities in patients with disabilities. This therapy allows for the extensive recovery of patients from injuries [32]. However, most studies on cognitive exercise therapy have been single-case or group studies, including non-randomized groups without a control group. Therefore, the present study evaluated the effects of cognitive exercise therapy with spatial and tactile tasks for the shoulder, elbow, and wrist joints on upper extremity function, sensory function, and daily activity compared to those of conventional occupational therapy in patients with stroke.

The results of the current study showed significantly improved daily activities, upper extremity motor function, and sensory function among patients in the cognitive exercise therapy group, while patients in the conventional occupational therapy group did not show significantly improved motor and sensory function, despite the significant group differences. Consistent with our findings, interventions involving tactile and spatial task training in cognitive exercise therapy improved upper extremity and sensory function [21,23,33]. Ahn (2009) performed training to perceive the hardness of a sponge on the shoulder joint of the affected upper extremity, as well as spatial tasks to improve direction in the shoulder, elbow, and wrist joints. The author reported significantly improved upper extremity function of the affected side compared to that of the unaffected side [23]. In addition, the cognitive exercise profile showed significantly improved direction of motion and awareness of the joint angles on the affected side. This is consistent with our finding of improved upper extremity motor and sensory functions, as well as significantly improved sensorimotor function, in the cognitive exercise therapy group. Furthermore, a previous study that investigated the effects of tactile and spatial cognitive training on the reduction in stiffness in hemiplegic patients reported that pressure awareness training of the elbow joint using sponges and spatial awareness training using a circular track plate reduced arm muscle stiffness by 23.3% and improved upper extremity function by 9.37% [33]. In addition, cognitive-motor profiling showed improved angle and spatial awareness in the shoulder, elbow, and wrist joints. Finally, in their study on hemineglect patients, Lim and Lee (2014) showed that cognitive exercise therapy led to positive improvements in upper extremity motor function and grip test results, suggesting the possible positive effects of cognitive exercise therapy on the rehabilitation of patients with stroke and hemineglect [34].

Although we observed no significant group difference in SFT, there was a significant group effect in tactile sensation among the sub-areas in the SFT. Previous research showed that cognitive exercise therapy improved tasks performed using spatial and tactile senses and that activating the premotor cortex areas, which supports the improvement in tactile sensory functions of the hand observed in the present study [35]. While upper extremity function improved due to the recovery of tactile sensation, the MFT did not differ significantly following conventional occupational therapy, as the upper extremities are the last to improve after stroke [36].

We observed significantly improved sensorimotor function after the intervention in the experimental group but not in the control group. However, daily life activity significantly improved after the intervention in both groups, although the two groups did not differ significantly. A study that applied spatial and tactile tasks to patients with stroke reported findings contrary to those of previous studies, in which daily life activity was significantly improved compared to the control group [37]. Consistent with our results, other studies have also reported functional improvement in the upper extremities and daily life activity performance after cognitive exercise therapy.

Therefore, to complement the limitations of previous studies, this study enrolled more participants and conducted a total of 20 cognitive exercise therapy sessions for 4 weeks, including an SFT, which is a key part of cognitive exercise therapy and is important for functional recovery in patients with stroke. Both cognitive exercise and conventional occupational therapies had positive effects on ADLs in patients with stroke. In contrast, sensorimotor function recovery was significantly improved only in the cognitive exercise therapy group, although significant differences were observed between the groups. Lee (2016) examined changes in electroencephalography (EEG), which records brain activities when healthy individuals distinguish between regular and irregular shapes, to show that tactile stimulation activated the parietal region of the brain following cognitive exercise therapy compared to conventional occupational therapy [38]. Instead, attention and perceptual awareness indicate changes in the central nervous system. In other words, when participants paid close attention to the body to interpret somatosensory information, attention, recognition, and cognition to solve spatial and tactile tasks led to EEG changes in the cerebral cortex, which mediates upper extremity functions. Cognitive exercise therapy is thought to improve body part recognition in patients with impaired upper extremity function due to stroke [39]. Considering the body as a surface receptor and transmission of delicate information to the brain by the body may have led to the superficial senses of touch and pressure as well as deep senses of joints and muscles affecting sensorimotor function and ADLs [39].

The results of this study showed that cognitive exercise therapy had positive effects on upper extremity function and sensorimotor function in patients with chronic stroke, while conventional occupational therapy did not, suggesting that the brain is highly activated when perceiving information or paying attention during the spatial and tactile tasks in the cognitive intervention [40]. Although the present study objectively examined the function of eight sensors using the SFT, several limitations must be considered when interpreting the findings. First, there were limitations in providing an optimal environment to achieve the full focus of the participants during the interventions. The participants were also not followed-up for post-intervention effects; thus, the long-term effects of cognitive exercise therapy could not be assessed. Additional studies are needed to confirm that cognitive exercise therapy is an effective treatment with potential for further development and implications. In addition, although validated measurement tools were used, the reliability of the test was not measured in the present study, which may mask the true effects of the intervention. Lastly, since the cognitive exercise group received both cognitive exercise and conventional occupational therapies, the intervention benefit cannot be assumed to be only due to cognitive exercise therapy; therefore, further studies should include a true experimental group receiving only cognitive exercise.

## 5. Conclusions

In conclusion, both groups showed improvement in daily activities after the intervention. Additionally, cognitive exercise therapy using spatial and tactile tasks significantly improved upper extremity motor and sensory functions compared to conventional occupational therapy. Therefore, cognitive exercise therapy compensates for the shortcomings of conventional occupational therapy used for rehabilitation and provides an effective rehabilitation protocol for patients with chronic stroke.

## Figures and Tables

**Table 1:** General participant characteristics (*n* = 30).

	EG (*n* = 15)	CG (*n* = 15)	*p*
Age (years)	62.5 ± 11.3	64.7 ± 14.3	0.633
Sex, Female, *n* (%)	6 (40.0)	11 (73.3)	0.139
Height (cm)	165.2 ± 7.7	160.5 ± 8.0	0.118
Weight (kg)	61.5 ± 7.1	59.6 ± 8.2	0.493
BMI (kg/m^2^)	22.5 ± 1.7	23.1 ± 2.2	0.456
Stroke type			
Infarction, *n* (%)	7 (46.7)	10 (66.7)	0.285
Hemorrhage, *n* (%)	8 (53.3)	5 (33.3)	
Affected side, left, *n* (%)	8 (53.3)	9 (60.0)	1.000
Onset (months)	19.9 ± 7.1	18.3 ± 7.0	0.540
K-MMSE (scores)	24.1 ± 3.8	23.9 ± 3.2	0.918

Abbreviations: EG—experimental group (cognitive exercise therapy); CG—control group (conventional occupational therapy); BMI—body mass index; K-MMSE—Korean version of Mini-Mental State Examination.

**Table 2:** Motor and sensory functions and daily activity before and after the interventions in the groups (*n* = 30).

Outcome Variables		EG (*n* = 15)	CG (*n* = 15)	Group × Time Interaction F(*p*)	Main Effect of Group F(*p*)
MFT	Pre-test	8.40 ± 9.34	8.80 ± 8.93	0.242 (0.626)	0.002 (0.961)
	Post-test	9.93 ± 9.53	9.87 ± 9.61		
	t(*p*) *	<0.001	0.251		
SFT	Pre-test	7.07 ± 2.91	8.20 ± 3.32	13.225 (0.001)	0.114 (0.738)
	Post-test	8.87 ± 2.97	8.47 ± 2.88		
	t(*p*) *	<0.001	0.104		
K-MBI	Pre-test	57.20 ± 19.27	46.73 ± 22.59	0.728 (0.401)	1.627 (0.213)
	Post-test	61.47 ± 20.64	53.07 ± 19.42		
	t(*p*) *	0.001	0.012		

Abbreviations: EG—experimental group (cognitive exercise therapy); CG—control group (conventional occupational therapy); MFT—manual function test; SFT—sensory function test; K-MBI—Korean version of modified Barthel index. * within-group effect.

## Data Availability

The datasets generated during the current study are available from the corresponding author upon reasonable request.

## References

[1] Lopez A.D., Mathers C.D., Ezzati M., Jamison D.T., Murray C.J. (2006). Global and regional burden of disease and risk factors, 2001: Systematic analysis of population health data. Lancet.

[2] Towfighi A., Saver J.L.J.S. (2011). Stroke declines from third to fourth leading cause of death in the United States: Historical perspective and challenges ahead. Stroke.

[3] Wolf S.L., Catlin P.A., Ellis M., Archer A.L., Morgan B., Piacentino A.J.S. (2001). Assessing Wolf motor function test as outcome measure for research in patients after stroke. Stroke.

[4] Pellegrino L., Coscia M., Muller M., Solaro C., Casadio M. (2018). Evaluating upper limb impairments in multiple sclerosis by exposure to different mechanical environments. Sci. Rep..

[5] Cirstea M., Ptito A., Levin M.F. (2003). Arm reaching improvements with short-term practice depend on the severity of the motor deficit in stroke. Exp. Brain Res..

[6] Doyle S., Bennett S., Fasoli S.E., McKenna K.T. (2010). Interventions for sensory impairment in the upper limb after stroke. Cochrane Database Syst. Rev..

[7] Patel N., Jankovic J., Hallett M.J.T.L.N. (2014). Sensory aspects of movement disorders. Lancet Neurol..

[8] Kamalakannan S., Chockalingam M., Sethuraman L., Moorthy S.D., Muthuvel T. (2021). Occupational Therapy for Reducing Disabilities in Persons with Disabilities in India: A Systematic Review. J. Occup. Ther..

[9] Schwartz J.H., Jessell T.M. (2000). Principles of Neural Science.

[10] Yoo-Soon B. (2007). The Effects of Task-Oriented Activities on the Cognitive Function and Performance of Activities of Daily Living in Stroke Patients. Korean J. Occup. Ther..

[11] Sabaté M., González B., Rodríguez M.J.N. (2004). Brain lateralization of motor imagery: Motor planning asymmetry as a cause of movement lateralization. Neuropsychologia.

[12] Jung J.H., Cho Y.N., Chae S.Y. (2011). The Effect of Task-Oriented Movement Therapy on Upper Extremity, Upper Extremity Function and Activities of Daily Living for Stroke Patients. J. Rehabil. Res..

[13] Jung M.Y., Chung B.I. (2009). The Effect of Constraint-induced Movement Therapy on the Affected Upper Extremity Function and Activities of Daily Living for Stroke Patients. Korean J. Occup. Ther..

[14] Sütbeyaz S., Yavuzer G., Sezer N., Koseoglu B.F. (2007). Mirror therapy enhances lower-extremity motor recovery and motor functioning after stroke: A randomized controlled trial. Arch. Phys. Med. Rehabil..

[15] Richardson A. (1967). Physical Education; Recreation. Mental practice: A review and discussion part I. Res. Q. Am. Assoc. Health Phys. Educ. Recreat..

[16] Aman J.E., Elangovan N., Yeh I.L., Konczak J. (2015). The effectiveness of proprioceptive training for improving motor function: A systematic review. Front. Hum. Neurosci..

[17] Takeuchi N., Izumi S.-I. (2013). Rehabilitation with poststroke motor recovery: A review with a focus on neural plasticity. Stroke Res. Treat..

[18] Franca P. (2018). Introduction to neurocognitive rehabilitation according to Carlo Perfetti. Vopr. Reabil..

[19] Jang J.-Y., Kim J.-M. (2012). The Effects of Cognitive Therapy Exercise on the Affected Upper Extremity Function and Activities of Daily Living for Stroke Patient. J. Korean Soc. Neurocogn. Rehabil..

[20] Lee S., Bae S., Jeon D., Kim K.Y. (2015). The effects of cognitive exercise therapy on chronic stroke patients’ upper limb functions, activities of daily living and quality of life. J. Phys. Ther. Sci..

[21] Jeong E.-M., Lee E.-M., Kim S.-G. (2016). The Effect of Motor Image on the Upper Extremity Function Recovery in Stroke Patients. J. Korean Soc. Neurocogn. Rehabil..

[22] Kim Y.-S. (2009). Effect of Cognitive Therapeutic Exercise on Improvement Hand Function with Hemiplegic Patient After Stroke. J. Korean Soc. Neurocogn. Rehabil..

[23] Ahn S.-N., Lee J.-W. (2009). Effect of Cognitive Therapeutic Exercise on Recovery of the Upper Limb Function in hemiplegia. J. Korean Soc. Neurocogn. Rehabil..

[24] Nakamura R., Moriyama S., Yamada Y., Seki K. (1992). Recovery of impaired motor function of the upper extremity after stroke. Tohoku J. Exp. Med..

[25] Miyamoto S., Kondo T., Suzukamo Y., Michimata A., Izumi S. (2009). Reliability and validity of the Manual Function Test in patients with stroke. Am. J. Phys. Med. Rehabil..

[26] Jung H.Y., Park B.K., Shin H.S., Kang Y.K., Pyun S.B., Paik N.J., Kim S.H., Kim T.H., Han T.R. (2007). Development of the Korean version of Modified Barthel Index (K-MBI): Multi-center study for subjects with stroke. J. Korean Acad. Rehabil. Med..

[27] Choi Y.-I., Kim W.-H., Park E.-Y., Kim E.-J. (2012). The validity, reliability and discriminative index of the Korean version of Modified Barthel Index (K-MBI) in stroke patients. J. Korea Acad. Coop. Soc..

[28] Choi J.-E., Lee S.-S. (2009). Effect of Stretch Reflex Control of the Upper Limb on Recovery Hand Function with Stroke Patient. J. Korean Soc. Neurocogn. Rehabil..

[29] Bier F.O., Fenn E.O., Kochobay A.O., Moran K.O., Naik K.O. (2014). Occupational Therapy Practice Framework: Domain and Process (3rd Edition). Am. J. Occup. Ther..

[30] Kuriakose D., Xiao Z. (2020). Pathophysiology and Treatment of Stroke: Present Status and Future Perspectives. Int. J. Mol. Sci..

[31] Raghavan P. (2015). Upper Limb Motor Impairment After Stroke. Phys. Med. Rehabil. Clin. N. Am..

[32] Perfetti C., Wopfner-Oberleit S. (1997). Der hemiplegische Patient: Kognitiv therapeutische Übungen.

[33] Kim M.-Y., Lee T.-Y. (2010). Effect of Tactile and Spacious Cognitive Task Training on Decrease of Spasticity With Hemiplegic Patient. J. Korean Soc. Neurocogn. Rehabil..

[34] Lim Y.-J., Lee S.-A. (2014). The Effect of Neurocognitve Rehabilitation on the Visual Perception and Upper-limb Function of Neglect. J. Korean Soc. Neurocogn. Rehabil..

[35] Van de Winckel A., Sunaert S., Wenderoth N., Peeters R., Van Hecke P., Feys H., Horemans E., Marchal G., Swinnen S.P., Perfetti C.J.N. (2005). Passive somatosensory discrimination tasks in healthy volunteers: Differential networks involved in familiar versus unfamiliar shape and length discrimination. NeuroImage.

[36] Fritz J., Shamma S., Elhilali M., Klein D. (2003). Rapid task-related plasticity of spectrotemporal receptive fields in primary auditory cortex. Nat. Neurosci..

[37] Kim Y. (2014). The Effectiveness of Cognitive Therapeutic Exercise on Upper Extremity Function and Activities of Daily living in Stroke Patients. J. Korean Soc. Neurocogn. Rehabil..

[38] Lee J., Jung B.R., Ahn S.N. (2016). The Difference of Electronencephalograph on Recognition of Formal and Geometric Shape in Adults. J. Korean Soc. Neurocogn. Rehabil..

[39] Hatem S.M., Saussez G., Della Faille M., Prist V., Zhang X., Dispa D., Bleyenheuft Y. (2016). Rehabilitation of Motor Function after Stroke: A Multiple Systematic Review Focused on Techniques to Stimulate Upper Extremity Recovery. Front. Hum. Neurosci..

[40] Andersson R., Mohme-Lundholm E. (1969). Studies on the relaxing actions mediated by stimulation of adrenergic alpha- and beta-receptors in taenia coli of the rabbit and guinea pig. Acta Physiol. Scand..

